# Application of the Subtractive Genomics and Molecular Docking Analysis for the Identification of Novel Putative Drug Targets against* Salmonella enterica* subsp.* enterica serovar* Poona

**DOI:** 10.1155/2017/3783714

**Published:** 2017-08-17

**Authors:** Tanvir Hossain, Mohammad Kamruzzaman, Talita Zahin Choudhury, Hamida Nooreen Mahmood, A. H. M. Nurun Nabi, Md. Ismail Hosen

**Affiliations:** Department of Biochemistry and Molecular Biology, University of Dhaka, Dhaka 1000, Bangladesh

## Abstract

The emergence of novel pathogenic strains with increased antibacterial resistance patterns poses a significant threat to the management of infectious diseases. In this study, we aimed at utilizing the subtractive genomic approach to identify novel drug targets against* Salmonella enterica* subsp.* enterica serovar* Poona strain ATCC BAA-1673. We employed in silico bioinformatics tools to subtract the strain-specific paralogous and host-specific homologous sequences from the bacterial proteome. The sorted proteome was further refined to identify the essential genes in the pathogenic bacterium using the database of essential genes (DEG). We carried out metabolic pathway and subcellular location analysis of the essential proteins of the pathogen to elucidate the involvement of these proteins in important cellular processes. We found 52 unique essential proteins in the target proteome that could be utilized as novel targets to design newer drugs. Further, we investigated these proteins in the DrugBank databases and 11 of the unique essential proteins showed druggability according to the FDA approved drug bank databases with diverse broad-spectrum property. Molecular docking analyses of the novel druggable targets with the drugs were carried out by AutoDock Vina option based on scoring functions. The results showed promising candidates for novel drugs against* Salmonella* infections.

## 1. Introduction 

Recent progress in the field of computational biology and bioinformatics has generated various in silico analysis and drug designing approaches, eliminating the time and cost involved in the trial and error experimentations that go into drug development [1]. These methods serve to shortlist the potential drug targets that will subsequently be used for laboratory testing. Subtractive genomics is one such in silico approach used for drug target identification based on determination of essential and nonhomologous proteins within the pathogenic organism [1, 2]. The Database of Essential Gene (DEG) server can be used for the identification of those proteins involved in important metabolic pathways required for the survival of the pathogen. Furthermore, determination of proteins homologous to humans can be screened out to avoid potential adverse drug reactions during the computer based drug development process. By selecting essential proteins unique to pathogen survival and propagation, the subtractive genomics technique allows identification of novel drug targets within the pathogen. Moreover in silico docking studies between the identified drug targets and existing drugs with slight modification may lead to the discovery of novel drugs for treatment of infection. As a result, a wide range of drug targets and lead compounds can be identified prior to laboratory experimentation, saving extensive time and money. This study focuses on identifying potential drug targets against* Salmonella enterica* subsp.* enterica serovar* Poona str. ATCC BAA-1673 using the subtractive genomics approach.

*Salmonella enterica* subsp.* enterica serovar* Poona str. ATCC BAA-1673 is a potent food-borne pathogen in humans [3].* Salmonella *belongs to the family of flagellated, Gram-negative, facultative anaerobic bacterium and is the causative agent of salmonellosis. In most people, infection by* Salmonella* is manifested as abdominal cramps, diarrhea, and fever which resolves itself in 4–7 days [4]. However, in some cases such as in infants, the elderly, and immune compromised patients, the* Salmonella* pathogen may penetrate the wall of the intestine and enter the circulation from which they can travel to other sites of the body. These cases have a high mortality rate and must be treated promptly with the use of antibiotics [4]. In 2015-2016, a total of 907 people were infected by* Salmonella *Poona in the United States alone [5]. Epidemiological and laboratory studies showed that this outbreak was transmitted through ingestion of contaminated cucumbers imported from Mexico [5]. In other cases, several separate outbreaks of salmonellosis have occurred due to exposure to pet turtles [6, 7]. Overall, salmonellosis incidence has not decreased in the past decade and while the incidences for some serotypes have decreased, incidences for some have increased, being attributed to the emergence of antibiotic resistant strains [8, 9]. Since 1996, several* Salmonella* serovars have started showing resistance to various antimicrobial agents, namely, ciprofloxacin and ceftriaxone [5]. Furthermore, CDC reported that 5% of* Salmonella* species are resistant to 5 or more types of drugs. Therefore, it is imperative to have a protective plan in case of major future outbreaks. Hence, we aim to apply the subtractive genomics approach to identify novel potential drug targets against* Salmonella enterica* subsp.* enterica serovar* Poona str. ATCC BAA-1673.

## 2. Methodology

### 2.1. Retrieval of Proteomes of Host and Pathogen

**Figure 1:**
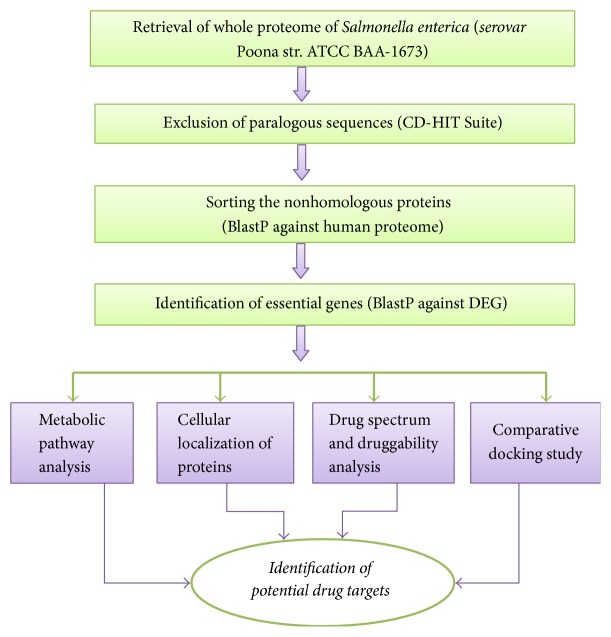
Schematic diagram of the flow chart for drug target identification.

The complete proteome of pathogen,* Salmonella enterica* subsp.* enteric serovar* Poona str. ATCC BAA-1673 (proteome ID: UP000017517), along with the complete proteome of the host,* Homo sapiens* (proteome ID: UP000005640) was retrieved from UniProt. The flow chart for different steps performed in the current study is given in Figure 1.

### 2.2. Identification of Essential Proteins in* Salmonella enterica* subsp. Poona

The whole proteome of* Salmonella enterica* subsp. Poona was subject to selective removal by CD-HIT Suite with the sequence identity cut-off at 60 [6] to remove all paralogous proteins within the pathogen. This filtered dataset was referred to as set 1 proteome, which was then used as the query sequence and subject to exclusion using BLAST+ 2.2.26 having a customized database for the human proteome with the cut-off expectation value (*E* – value) of 10^−4^ to acquire set 2 proteome dataset which did not contain any homologous proteins to those of* Homo sapiens*. BlastP analysis was carried out for set 2 proteome using DEG in which genes indispensable for the survival of* Salmonella* genus were selected as the reference database. *E*-value cut-off score less than 10^−100^ and a minimum bit score cut-off of 100 were used to obtain a set of essential genes. Thus, the resulting protein sequences (set 3 proteome) obtained were nonhomologous to* Homo sapiens* proteome and represent a way to subtract the host proteome from further analysis.

### 2.3. Analyses of Metabolic Pathway(s)

The essential proteins of* Salmonella enterica serovar* Poona as identified above was subject to metabolic pathway analysis using KAAS (KEGG Automatic Annotation Server) at KEGG for the identification of potential targets. KAAS (KEGG Automatic Annotation Server) provides functional annotation of genes by BLAST comparisons against the manually curated KEGG GENES database. The result contains KO (KEGG Orthology) assignments and automatically generated KEGG pathways [10].

### 2.4. Prediction of Subcellular Location

The subcellular locations of the essential proteins must be known for determination of suitable drug targets by allowing prediction of protein function and genome annotation. Computational prediction methods are used to establish the location of a particular protein in the cell. PSORTb version 3.0 (http://www.psort.org/psortb/) was used for this purpose. CELLO version 2.5 (http://cello.life.nctu.edu.tw/) [11] was used to cross-check the data obtained from PSORTb. The proteins were then sorted according to their subcellular localization.

### 2.5. Evaluation of Druggability Potential of the Essential Proteins

The essential proteins associated with the unique pathway in* Salmonella enterica *were subject to BlastP analysis against the customized database that is retrieved from drug bank for all FDA approved drug targets [12]. Targets that showed highly matched frequency (80% or more) with database for FDA approved drugs are druggable target. On the other hand, targets that did not show considerable degree of matching with the FDA approved drugs are considered as the novel target for new drug identification.

### 2.6. Analysis of Drug Spectrum

BlastP was performed individually for each of the drug targets found above against a database containing nonredundant protein sequences. As obtained from the taxonomy report, if the drug targets were found to be present in greater than 25 bacteria, they were classified as broad-spectrum targets. Different bacterial species were used as references.

### 2.7. Molecular Docking Analysis of the Novel Target with the Drugs

In order to understand the structural basis of the protein targets specificity with the drugs, a computational target-ligand docking approach was used to analyze structural complexes of the novel druggable targets with the ligands (drugs). For this purpose, the three-dimensional structures of the novel druggable targets were downloaded from the UniProt database. The chemical structures of the ligands were obtained from DrugBank database [12]. For docking analysis, coordinates of the target protein and potential drug molecule were optimized by Drug Discovery Studio version 3.0 software and UCSF Chimera tool, respectively. Molecular docking analyses of the druggable targets with the drugs were carried out by AutoDock Vina option based on scoring functions [13]. The energy of interaction of the ligands with the targets is assigned “grid point.” The grid box was optimized to cover the whole area of the target.

## 3. Results and Discussion

This article describes a simple subtractive genomics approach for identification of a suitable drug target among the essential proteins within the proteome of* Salmonella enterica serovar* Poona. The subtractive genomics approach has been reported as an innovative and powerful method for identifying unique sequences as potential therapeutic targets [2, 14, 15]. The in silico subtractive genome analysis is based on sorting the essential proteins of a pathogen as unique (absent in the host organism) in order to facilitate precise drug designing by avoiding host toxicity through cross-reactivity with* Homo sapiens *proteome.

### 3.1. Identification of Nonhomologous Essential Proteins

*Salmonella enterica serovar* Poona contains a total of 4906 proteins in its proteome. Following analysis with CD-HIT suite, 154 proteins were found to be duplicates or paralogs with 60% identity and were eliminated from the dataset as these were redundant as drug targets. The remaining 4752 proteins (set 1 proteome) were analyzed using BlastP against a customized human protein database and 1088 proteins were found to be homologous to human proteins and were again excluded from the dataset as these proteins may cause drug cross-reactivity and host cytotoxicity when used as drug targets during treatment. The resulting set 2 proteome containing 3664 proteins was used for further analysis and subject to BlastP search in the database of essential genes (DEG) in order to determine the essential genes required for the survival of the pathogen. A total of 198 proteins were found to be essential, which means that these proteins are involved in metabolic pathways indispensable for the propagation of this pathogen and thus can be used as target for treatment options (Table 1).

### 3.2. Metabolic Pathway Analysis

The set of 198 proteins deemed to be essential through the DEG analysis was passed through the KEGG-KASS server to analyze their metabolic pathway. It was found that 52 proteins were involved in metabolic pathways unique to the* Salmonella enterica* species and thus, not found in humans. The associated pathways along with the names of essential genes and their KO have been presented in Table 2.

A metabolic pathway of particular importance is the lipopolysaccharide biosynthesis in* Salmonella* Poona. LPS is composed of a conserved core oligosaccharide, lipid A, linked to a variable O-antigen in the cell membrane of the Gram-negative bacteria, thus, providing outer membrane stability [16]. 2-Dehydro-3-deoxyphosphooctonate aldolase (KDO 8-P synthase) was recognized as a potential drug target specific to this pathway.

Peptidoglycan composes the cell wall of bacterial cells and inhibitors of peptidoglycans form a major class of antibiotics. Drug targets that inhibit peptidoglycan biosynthesis can minimize microbe generated pathogenicity [14]. Three unique proteins involved in peptidoglycan biosynthesis within* Salmonella* Poona species were found to be inhibited by drugs, namely, alanine racemase, UDP-N-acetyl glucosamine 1-carboxyvinyltransferase and penicillin–binding protein 2.

Two-component system is a signal transduction system responsible for sensing any change in the environment or intracellular state of the bacteria and inducing the appropriate response to adapt to these changes [17, 18]. Thus, proteins involved in this pathway are better drug targets and their inhibition will make bacteria susceptible to various drugs. Using current in silico approaches, four such proteins were found and they are outer membrane channel protein tolC, outer membrane pore protein F, histidine kinase PhoQ, and histidine kinase envZ.

Cationic antimicrobial peptides (CAMPS) are key components of the innate immune system and weaken the bacterial cell membrane integrity. On the other hand, various bacteria, including* Salmonella *Poona, have developed pathways that attribute resistance to CAMP [19, 20]. Metabolites involved in this pathway are good targets for altering CAMP resistance and cancelling virulence. Our study found several target molecules, which may interfere with the pathways responsible for developing resistance.

Vancomycin is a glycopeptide antibiotic which is active against most Gram-positive bacteria. This inhibits the synthesis of peptidoglycan in the bacterial cell walls by interacting with D-Ala-D-Al-pentapeptide at C-terminus and preventing their addition to the peptidoglycan chain [21].

### 3.3. Druggability of the Unique Essential Proteins

Since the ultimate goal of the current study was to identify novel drug targets, the next step was to evaluate the druggability of the essential proteins that were involved in unique* Salmonella* specific metabolic pathway. The analysis of druggable targets, available drugs, and broad-spectrum property of the drugs showed that 11 of the shortlisted unique essential proteins are druggable according to the FDA approved DrugBank databases with diverse broad-spectrum property (Table 3).

### 3.4. Novel Druggable Targets

The proteins in Table 3 represent the already identified drug targets with FDA approved drugs. In order to identify the novel druggable targets among the shortlisted protein sequences, we further carried out BLAST analysis of the essential proteins against the DrugBank database. A total of 6 different proteins selected from the 52 proteins that are associated with unique pathway were identified to be plausible novel targets. These proteins were chosen on the basis of their uniqueness and essentiality in pathogen-specific vital pathways. All the 52 proteins that were presented in Table 2 are essential for the existence of the specific* Salmonella* strain which was identified via KEGG pathway analysis. Among these, the druggable 11 proteins presented in Table 3 showed more than 80% identical similarity with the already FDA approved drug targets. But there were also other 41 proteins left in the unique protein groups for this strain presented in Table 3. Inhibition of these 41 proteins also can be used to fight against this specific microbe and these 41 also showed to some extent identical similarity (<80%) with the targets of FDA approved drugs that were used against other organisms. As it has been known, drug molecule does not bind with the whole protein to perform its activity. Amino acid sequences of the drug binding active site of the whole protein are the important residues for the binding of a drug to a protein. Among these 41 proteins, 6 were presented in Table 4. The reasons behind choosing these 6 proteins were as follows: (A) these proteins showed moderate similarity (65–30%) with the targets of FDA approved drugs that were used against other organisms. As it has been known that amino acid sequences of the drug binding active site of the whole protein are the important residues for the binding of a drug to a protein, these 65–30% identical residues may be laid within the drug binding active site residues. (B) These 6 proteins were also unique to this specific* Salmonella* strain and associated with the essential pathways which are important for the existence of this organism. UDP-N-acetylglucosamine O-acyltransferase is associated with both lipopolysaccharide biosynthesis and cationic antimicrobial peptide (CAMP) resistance pathways. UDP-3-O-[3-hydroxymyristoyl] N-acetyl-glucosamine-deacetylase and 3-deoxy-manno-octulosonate cytidylyltransferase (CMP-KDO synthetase) are associated with lipopolysaccharide biosynthesis pathway. Phosphate regulon response regulator OmpR, nitrogen regulation sensor histidine kinase GlnL, and response regulator CheB are associated with two-component system pathway. LPS is composed of a conserved core oligosaccharide, lipid A, linked to a variable O-antigen in the cell membrane of the Gram-negative bacteria, thus providing outer membrane stability [16]. Drug that inhibits lipopolysaccharide (LPS) biosynthesis can kill the microbe. Cationic antimicrobial peptides (CAMPs) are key components of the innate immune system and weaken the bacterial cell membrane integrity. On the other hand, various bacteria, including* Salmonella* Poona have developed pathways that attribute resistance to CAMP [19, 20]. Metabolites involved in this pathway are good targets for altering CAMP resistance and cancelling virulence. Two-component system is a signal transduction system responsible for sensing any change in the environment or intracellular state of the bacteria and inducing the appropriate response to adapt to these changes [17, 18]. Thus, proteins involved in this pathway are better drug targets and their inhibition will make bacteria susceptible to various drugs.

### 3.5. Molecular Docking of the Novel Druggable Proteins

**Figure 2:**
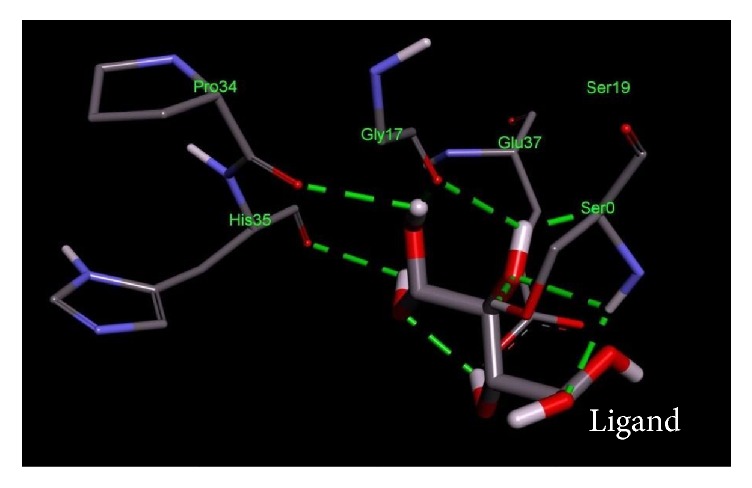
Important residues of the binding site of UDP-N-acetyl glucosamine O-acyltransferase of* Salmonella enterica *subsp.* enterica serovar *Poona observed to be interactive with the D-tartaric acid as ligand.

**Figure 3:**
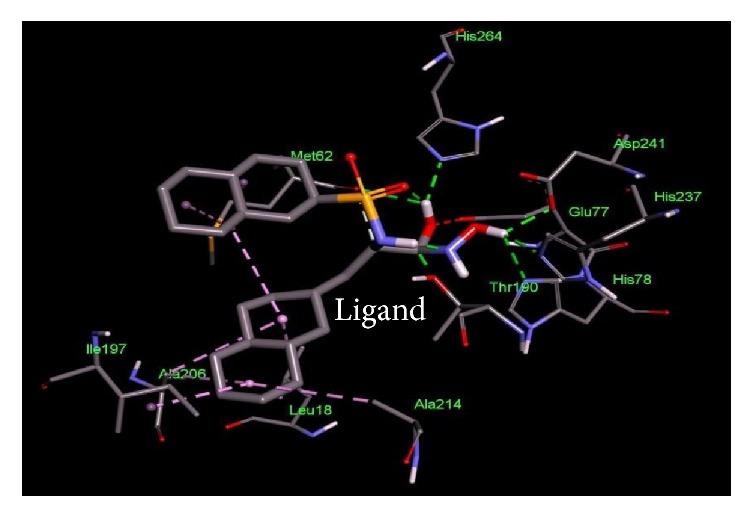
Important residues of the binding site of UDP-3-O-[3-hydroxymyristoyl] N-acetyl glucosamine deacetylase of* Salmonella enterica *subsp.* enterica serovar *Poona observed to be interactive with the (2R)-N–hydroxy–3–naphthalen–2-yl-2-[(naphthalen-2ylsulfonyl)amino]propanamideas ligand.

**Figure 4:**
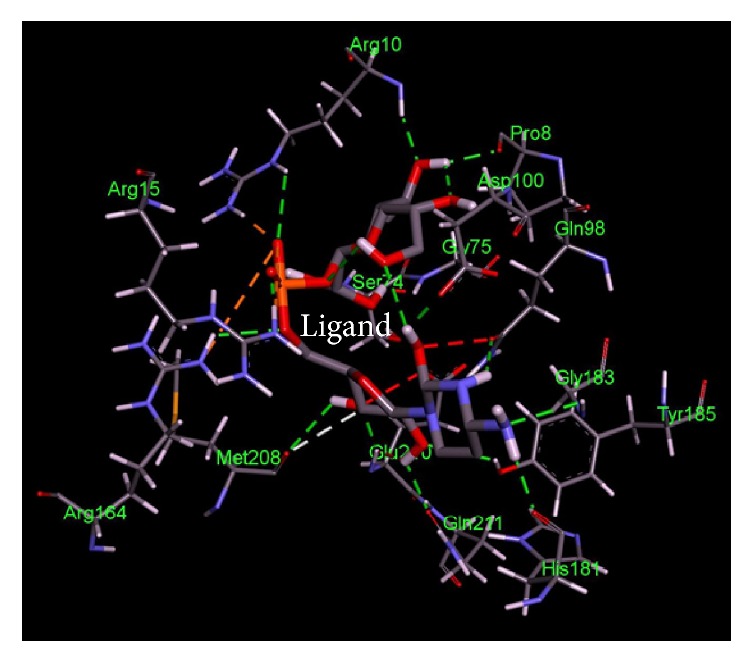
Important residues of the binding site of 3-deoxy-manno-octulosonate cytidylyltransferase of* Salmonella enterica *subsp.* enterica serovar *Poona observed to be interactive with the Cmp-2-Keto-3-Deoxy-Octulosonic Acid as ligand.

**Figure 5:**
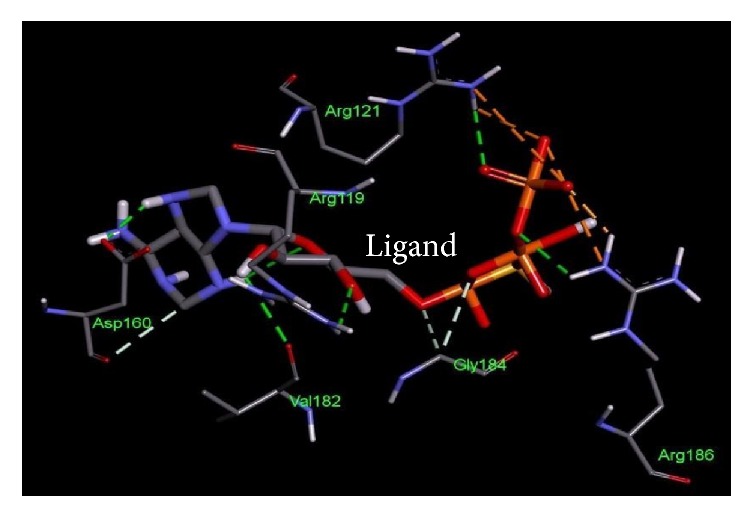
Important residues of the binding site of phosphate regulon response regulator OmpR of* Salmonella enterica *subsp.* enterica serovar *Poona observed to be interactive with the heparin disaccharide Iii-Sas ligand.

**Figure 6:**
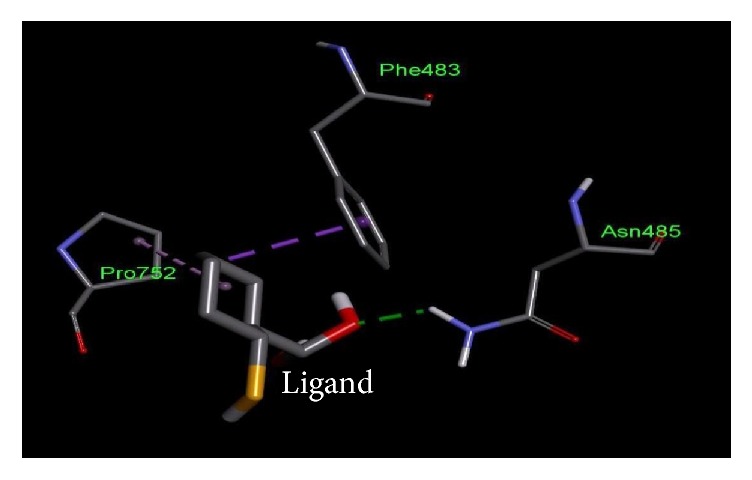
Important residues of the binding site of nitrogen regulation sensor histidine kinase GlnL of* Salmonella enterica *subsp.* enteric serovar *Poona observed to be interactive with the ethylmercurithiosalicylic acid as ligand.

**Figure 7:**
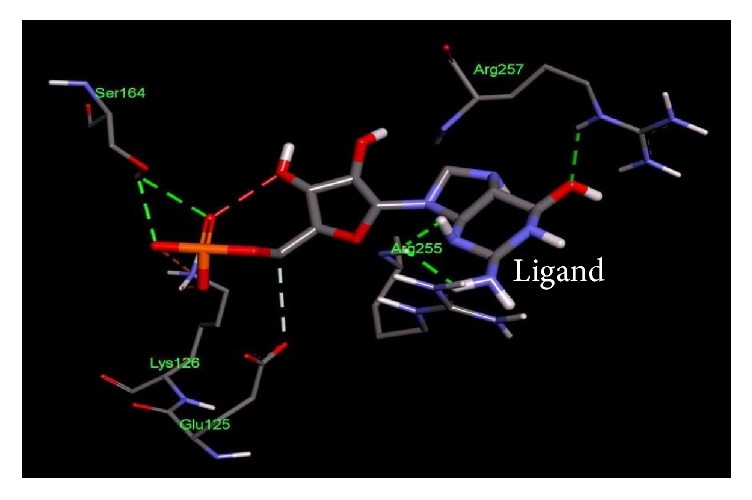
Important residues of the binding site of chemotaxis family, response regulator CheB of* Salmonella enterica *subsp.* enterica serovar *Poona observed to be interactive with the Guanosine-5′-Monophosphate ligand.

The current study was further reinforced by performing comparative docking studies of the novel druggable proteins with the ligands. Binding affinities from docking were compared between our target proteins and intended targets from other species against the corresponding drug. The shortlisted potential drug targets showed a pattern of similar binding characteristics, similar residues involved in the active site, and lower free energy (Table 4 and Figures 2–7). Thus, the potential targets with similar binding affinities to the intended proteins-drug affinities can be deemed as novel drug targets to be used in treatment strategies.

## 4. Conclusion

The vast array of information regarding the proteomes and genomes of various prokaryotic organisms and knowledge obtained from the human genome project can be manipulated to accelerate drug designing and gain further knowledge of pharmacogenomics in the treatment of bacterial infections. Subtractive genomics can aid in the identification of proteins targeted by existing FDA approved targets. A total of 52 potential targets were found within the* Salmonella* Poona system. Among these, 11 proteins were already highly identical with the FDA approved drug targets. 6 proteins were proposed as novel drug targets to combat against* Salmonella* Poona which showed moderate similarity (65–30%) with the targets of FDA approved drugs that were used against other organisms. Furthermore, docking studies were used to predict the binding of existing FDA approved drugs to the novel targets within the proteome of the pathogen and also with the drug-specific FDA approved database target to compare the binding pattern between them.

## Figures and Tables

**Table 1:** Subtractive proteomic and metabolic pathway analysis result for *Salmonella enterica *subsp.* enterica serovar *Poona.

Features of *Salmonella enterica *subsp.* enterica serovar *Poona	Number
Total number of proteins	4906
Duplicates (*>*60% identity) in CD-HIT	154
Nonparalogs	4752
Nonhuman homologous proteins (*E*-value 10^−4^)	3664
Essential protein in DEG (*E*-value 10^−100^)	198
Pathways unique to *Salmonella enterica *subsp.* enterica serovar *Poona	14
Proteins involved in unique pathways	52

**Table 2:** Essential proteins of *Salmonella enterica *subsp. *enterica serovar *Poona involved in unique metabolic pathways.

Associated pathway	Gene(s) name	KEGG orthology (KO)
D-Alanine metabolism	Alr	K01775

Vancomycin resistance	alr, murG	K01775, K02563

Lipopolysaccharide biosynthesis	lpxA, lpxC, lpxD, lpxH, lpxB, lpxK, kdtA, lpxL, kdsB, kdsA, lpxM, waaQ, waaC, waaF, waaG, waaJ,	K00677, K02535, K02536, K03269, K00748, K00912, K02527, K02517, K00979, K01627, K02560, K02849, K02841, K02843, K02844, K03279,

Cationic antimicrobial peptide (CAMP) resistance	lpxA, tolC, phoQ, arnT, pmrK, acrB, mexB, adeJ, smeE, mtrD, cmeB	K00677, K12340, K07637, K07264, K18138

Peptidoglycan biosynthesis	murA, murC, murD, murF, mraY, murG, murJ	K00790, K01924, K01925, K01929, K01000, K02563, K03980, K05515

Cell cycle	murG, dnaA, dnaB, ftsZ, ftsQ, ftsA	K02563, K02313, K02314, K03531, K03589, K03590

Bacterial secretion system	tolC, yscJ, sctJ, hrcJ, yscT, sctT, hrcT, secD, secY, secA, tatC	K12340, K03222, K03228, K03072, K03076, K03070, K03118

Two-component system	tolC, phoQ, envZ, ompR, ompF, rcsB, dnaA,glnL, ntrB,cheB, pagO	K12340, K07637, K07638, K07659, K09476, K07687, K02313, K07708, K03412, K07790

Protein export	secD, secY, secA, tatC	K03072, K03076, K03070, K03118

DNA replication	dnaB	K02314

Bacterial chemotaxis	cheB	K03412

**Table 3:** Druggable targets, available drugs, and broad-spectrum property analysis of the shortlisted essential proteins from *Salmonella enterica *subsp.* enterica serovar *Poona.

Target number	KEEG orthology (KO)	Protein name	Broad-spectrum property	Available drug in DrugBank	DrugBank IDs
(1)	K01775	Alanine racemase	25	Cycloserine	DB00260

(2)	K00790	UDP-N-Acetyl glucosamine 1-carboxyvinyltransferase	19	Fosfomycin	DB00828

(3)	K05515	Penicillin-binding protein 2	8	Ceftazidime Ertapenem	DB00438 DB00303

(4)	K12340	Outer membrane channel protein tolC	28	Colistin	DB00803

(5)	K09476	Outer membrane pore protein F	26	Polymyxin B Sulfate	DB00781

(6)	K03531	Cell division protein FtsZ	29	Guanosine 5′-diphosphate	DB04315

(7)	K01627	2-Dehydro-3-deoxyphosphooctonate aldolase (KDO 8-P synthase)	8	2-Phosphoglyceric acid	DB01709

(8)	K02563	UDP-N-Acetylglucosamine--N-acetylmuramyl-(pentapeptide) pyrophosphoryl-undecaprenol N-acetylglucosamine transferase	29	Uridine diphosphate-N-acetylgalactosamine	DB02196

(9)	K07637	Two-component system, OmpR family, sensor histidine kinase PhoQ	197	Radicicol	DB03758

(10)	K07638	Two-component system, OmpR family, osmolarity sensor histidine kinase EnvZ	32	Phosphoaminophosphonic acid-adenylate ester	DB04395

(11)	K18138	Multidrug efflux pump arcB	279	Rhodamine 6g	DB03825

**Table 4:** Lowest docking energies and important residues of the binding site observed to be interactive with the ligands.

Protein name or names of molecules	UDP-N-Acetylglucosamine O-acyltransferase	UDP-3-O-[3-Hydroxymyristoyl] N-acetyl glucosamine deacetylase	3-Deoxy-manno-octulosonate cytidylyltransferase	Phosphate regulon response regulator OmpR	Nitrogen regulation sensor histidine kinase GlnL	Chemotaxis family, response regulator CheB
UniProt ID of the database target	O25927	P47205	P44490	O32393	Q9X180	Q9A5I5

% identity	42.688	57.237	63.855	39.516	30.00	37.273

Possible drug	D-Tartaric acid.	(2R)-N-Hydroxy-3-naphthalen-2-yl-2-[(naphthalen-2-ylsulfonyl)amino]propanamide	Cmp-2-Keto-3-Deoxy-Octulosonic Acid	Heparin disaccharide Iii-S	Ethylmercurithiosalicylic acid	Guanosine-5′-Monophosphate

DrugBank ID	DB01694	DB07861	DB04482	DB02353	DB02731	DB01972

Being used against	*Helicobacter pylori*	*Pseudomonas aeruginosa*	*Haemophilus influenzae*	*Arthrospira platensis*	*Thermotoga maritima*	*Caulobacter crescentus*

Binding affinity of the drug with potential target (kcal/mol)	−4.4	−9.8	−10.4	−9.0	−4.8	−7.2

Binding affinity of the drug with database target Kcal/mol	−4.8	−9.2	−8.2	−9.9	−4.3	−6.2

Associated amino acid residues present in the database target binding pocket	Gly(48), Gly(65), Glu(76)	Leu(18), Met(62), Glu(77), His(78), Thr (190), Ile(197), Ala(206), Ala(214), His(237), Asp(241), His(264)	Gly(75), Thr(76), Asn(96), Gln(98), His(185), Gly(187), Tyr(189), Leu(213), Glu(214), Gln(215)	Asp(1017), Phe(1021), Thr(1022), Val(1059), Glu(1153)	Asp(464), Glu(467), Tyr(487), Phe(489)	Asp(41), Arg(114), Gly(137), Tyr(143), Glu(144)

Associated amino acid residues present in the potential target binding pocket	Ser(0), Gly(17), Ser(19), Glu(37), Pro(34), His(35)	Leu(18), Leu(62), Glu(78), Phe(161), Phe(192), Phe(194), Ile(198), Cys(207), Ala(215), His(265)	Pro(8), Arg(10), Arg(15), Ser(74), Gly(75), Gln(98), Asp(100), Arg(164), His(181), Gly(183), Tyr(185), Met(208), Glu(210), Gln(211)	Arg(119), Arg(121), Asp(160), Val(182), Gly(184), Arg(186)	Phe(483), Asn(485), Pro(752)	Glu(125), Lys(126), Ser(164), Arg(255), Arg(257)
